# Physiotherapy Strategies for Redefining Recovery in a Patient With Grade II Ependymoma: A Case Report

**DOI:** 10.7759/cureus.58809

**Published:** 2024-04-23

**Authors:** Krutika Dhawde, Lajwanti Lalwani, Anam R Sasun

**Affiliations:** 1 Department of Neurophysiotherapy, Ravi Nair Physiotherapy College, Datta Meghe Institute of Higher Education and Research, Wardha, IND; 2 Department of Cardiovascular and Respiratory Physiotherapy, Ravi Nair Physiotherapy College, Datta Meghe Institute of Higher Education and Research, Wardha, IND

**Keywords:** physiotherapy, case report, neuro-physiotherapy rehabilitation, quality of life, tardieu scale, functional independence measure, integrative approach

## Abstract

Neuroepithelial tumors known as ependymomas can develop from cortical rests, the central canal of the spinal cord, or the ependymal cells of the cerebral ventricles. Ependymomas may arise anywhere along the neuraxis. Here, we present a 40-year-old male, a known case of grade II ependymomas, with a chief complaint of bilateral lower limb weakness and loss of sensation in the bilateral lower limb for 20 days. He started facing difficulties in performing activities such as walking, toileting activities, and squatting activities. The physiotherapy (PT) rehabilitation of the patient was tailored to achieve functional independence of the patient. The treatment session lasted for six weeks. Several outcome indicators were employed to evaluate our patient's progress toward functional recovery. Outcomes are measured using the Tone Grading Scale (TGS), the American Spinal Injury Association (ASIA) Impairment Scale, the World Health Organization Quality of Life (WHOQOL), manual muscle test, and the Barthel Index. Outcome measures were assessed on day one of treatment and the last day of the PT treatment. The patient's preliminary involvement in PT supported him to prevent serious complications like joint contractures and bed sores. Physical therapy is one of the most important parts of the rehabilitation practice for spinal cord injury (SCI) patients.

## Introduction

Glial cell tumors, known as ependymomas, typically develop in the ventricular system's lining cells. Still, they can also occur outside of the central nervous system (CNS) or in the brain parenchyma [1]. Grades I-II tumors are gliomas that grow slowly. They more frequently affect children than adults and are made up of genetically unique subgroups of tumors [2]. Rare tumors of neuroectodermal origin and ependymomas are categorized as grade I, II, and III anaplastic ependymoma, myxopapillary ependymoma, and sub-ependymoma. The more common location is infratentorial (60%) [3]. The literature on adult intracranial ependymomas is limited due to the low incidence. Most series combine grade II and grade III tumors, as well as pediatric and adult ependymomas [4].

Additionally, the series are retrospective, include a small number of patients, and span several decades, during which time therapeutic and diagnostic modalities have changed [5]. Common signs and symptoms of spinal cord compression in patients with grade II ependymoma include paresthesia, gait ataxia, lower extremity spasticity, and sensory loss [6]. In a more advanced stage of the disease, a lumbar tumor causes a significant mass effect and can cause asymmetric weakness, radicular back and leg pain, and bladder incontinence [1]. Whereas some standardized assessments of impairments are specific to spinal cord injury (SCI), most are comparable to those used in all additional areas of physiotherapy (PT). For example sensation assessments, for instance, are done specifically for SCI and in accordance with the International Standards for Neurological Classification of SCI [7].

PT interventions are commonly employed to enhance the voluntary strength of muscles that are neurologically weak due to SCI. PT interventions that frequently serve this purpose include hand therapy, electrical stimulation, resistance training, and a variety of gait training interventions [8]. Strength maximizes function following SCI, so it is critical to comprehend how these interventions affect it. To improve stability, a wide range of interventions are suggested and put into practice. For many individuals with SCI, incontinence persists despite a range of available treatments, including conservative therapies. Strengthening the pelvic floor muscles (PFM) and controlling the bladder sphincter can be accomplished through PFM training, which is achieved by Kegel's exercise [9]. The prognosis for high-grade ependymomas in the spine is significantly worse than that of lower grades.

The principles governing physical therapy rehabilitation for people with SCIs are described in this report, along with the data supporting the efficacy of popular physical therapy treatments. It focuses on three common issues: poor motor control, contractures, and weakness. This report focuses solely on the rehabilitation stage, but physiotherapists are crucial both in the immediate aftermath of an injury and in the community after a patient leaves the hospital. In the course of the rehabilitation stage, the goals of physical therapy are centered around motor tasks like walking, pushing a wheelchair, transferring, and using the upper limbs [10]. This case report aims to describe the PT functional recovery of a patient with non-traumatic SCI, particularly grade II ependymomas with cord compression.

## Case presentation

Patient information

A 40-year-old man had presented to the Neuro-Rehabilitation Center with complaints of bladder dysfunction and bilateral lower limb weakness for the past 20 days. The weakness had gradually progressed and had begun to affect his daily activities, including standing, walking, and using the toilet. Magnetic resonance imaging (MRI) revealed a hyperintense mass at 1T5, T6, and T7 levels, suggestive of Grade II spinal ependymoma and spinal cord compression. Spinal cord compression and Grade II ependymoma were observed at the T5 and T6 levels. The patient had undergone both neuro-rehabilitation and chemotherapy sessions. Upon sensory examination, neither deep nor superficial sensations were present in either lower limb. Based on the non-traumatic American Spinal Injury Association (ASIA) Impairment Scale, the neurological level of sensations had been determined to be T5-T6. Following motor examination, atrophy of the lower limb muscles had been noted, with no power and weakness in his lower limb, rated Grade 0 on the Oxford Scale. The muscle tone was assessed by a Tone Grading Scale (TGS), and the findings revealed decreased tone in bilateral lower limbs, The reflex examination revealed absent reflexes in bilateral lower limbs as demonstrated in Table 1. The patient also demonstrated a foot drop in the bilateral foot. 

**Table 1:** Describing muscle tone and reflex examination (pre-rehabilitation)

Muscle tone	Right	Left
Hip flexors	+1	+1
Knee flexors	+1	+1
Ankle plantarflexors	+1	+1
Ankle dorsiflexors	+1	+1
Lower limb reflexes	Right	Left
Knee jerk	absent	absent
Ankle jerk	absent	absent
Plantar	absent	absent

Therapeutic interventions

The therapeutic interventions were designed according to the PT assessment done and according to the problem listing done by treating therapist. The PT rehabilitation focused on preventing secondary complications for the patient; it also focused on normalizing the tone of the muscles and strengthening PFM to improve urinary incontinence along with other interventions (Table 2). 

**Table 2:** Summarization of neuro-rehabilitation given to the patient D1-D2: diagonal pattern

Problem identified	Interventions
Preventions of secondary complications such as the formation of bed sores and thrombosis	Change in position every two hours.
Patient and relatives unaware of the prognosis/recovery of the patient	Patient and therapist rapport building, making patient and family aware of the condition and prognosis of it.
Unable to perform bed transfers independently	Bed-mobility exercises (log-rolling and supine-side-lying transfer).
Bladder incontinence	Kegel’s exercises, contraction of transverse abdominis, and hip abductor-adductor roll.
Bilateral foot drop	Functional electrical stimulation for foot plantar flexors and dorsiflexors (30 repetitions, five sets).
Flaccidity of bilateral lower limbs	Rood’s facilitatory approach.
Weakness of trunk and pelvis muscles	Trunk and pelvis proprioceptive neuromuscular facilitation exercises (20 repetitions, five sets), crunches exercises (straight, diagonal), and weight shift exercises in a quadruped position.
To improve static balance	Pelvis scooting exercises in a sitting position, multiplanar task-reach-out activities.
Sensory integration	Using different textures such as feathers, wool, silky, cotton, and sand.
Reduced air entry	Breathing exercises like thoracic expansion and diaphragmatic exercises were commenced.
To maintain strength of the bilateral upper limb	Proprioceptive neuromuscular facilitation (D1-D2 flexion, extension), using red Theraband.

Outcome measures

The patient underwent PT rehabilitation for seven weeks, six days/week. The outcome measures of the rehabilitation are described in Table 3. The post-rehabilitation findings of tone and reflexes are described in Table 4.

**Table 3:** Outcome measures of the patient ASIA: American Spinal Injury Association; WHOQOL: World Health Organization Quality of Life Scale

Outcome measures	Day 1	Last day
ASIA Impairment Scale	Motor score: upper extremity score: 40/50, lower extremity score: 0/50	Motor score: upper extremity score: 40/50, lower extremity score: 20/50
	Sensory score: light touch total: 17/56, pinprick total: 34 /112	Sensory score: light touch total: 30/56, pinprick total: 60/112
WHOQOL	40/100	60/100
Barthel Index	20/100	40/100

**Table 4:** Post-rehabilitation findings of tone and reflexes

Muscle tone	Right	Left
Hip flexors	+2	+2
Knee flexors	+2	+2
Ankle plantarflexors	+2	+2
Ankle dorsiflexors	+2	+2
Reflex	Right	Left
Knee jerk	1+	1+
Ankle jerk	1+	1+

## Discussion

There being a better understanding of the secondary mechanisms of SCI, new approaches to improving patient outcomes have been developed [11]. The term "activity-based therapy" has recently gained prominence due to its emphasis on neural plasticity and neural recovery after the compression of the spinal cord [10]. Some have praised activity-based therapy as an innovative approach to spinal cord compression PT [12]. Activity-based therapy bases a key element of context- and task-specific intensive practice involving multiple hours of exercise per day on the suggestions of Carr and Shepherd from the 1980s [13].

Strength training, treadmill or robotic walking with or without electrical stimulation, and "developmental sequencing" exercises are also encompassed [14]. According to reports, this specific form of therapy differs greatly from "conventional" or "traditional" therapy, where some practitioners only use compensatory methods devoid of providing therapeutic care below the injury level. Anecdotal evidence indicates that this is a false dichotomy and that long before activity-based therapy gained widespread acceptance, physiotherapists were providing therapeutic care below the level of injury, albeit mostly to patients who showed at least some signs of sensory and motor function [11]. According to the terminology, at least one trial has produced evidence that, after the spinal cord compression, individuals with grade II ependymomas benefit from intense PT in terms of improved motor and sensory function with an increase in strength. Some state that this affirms an entirely new kind of treatment, but others think the treatment in this report is no different from the treatment people with these kinds of lesions have been receiving for many years. As such, the trial provides much-needed evidence of the therapeutic benefits of a comprehensive and rigorous PT program.

Throughout the following decade's time, PT practice may undergo significant change. People who are paralyzed in their lower limbs can now walk over the ground as a result of exoskeletons [15]. Individuals with spinal cord compression may also benefit in the foreseeable future from stem cell therapy. Despite this, the report still needs to focus on some of the basic ideas that guide the management of PT patients with spinal cord compressions [16]. For instance, additional clinical trials need to be performed to assess the efficacy of commonly prescribed therapies for the management of various impairments, such as respiratory compromise, contracture, osteoporosis, weakness, and spasticity [17]. The subsequent advances in neuroplasticity, robotics, stem cell therapy, and other fields may require an adequate basis of evidence and knowledge of the most effective ways to treat these primary impairments [18]. It is imperative that future interventions are appropriately scrutinized within clinical trials before being implemented and established as standard practice [19]. For those concerned with spinal cord compression symptoms such as motor and sensory dysfunctions, evidence-based PT must be determined by high-quality studies [20].

## Conclusions

Non-traumatic SCI secondary to ependymomas negatively impacts the quality of life of patients. PT rehabilitation works to enhance the standard of life of the patient by enhancing the functional recovery of the patient. The designed protocol paves a pathway for future large-scale studies in SCI patients. The interventions that were planned for the patient according to the patient's problems, such as bed-mobility exercise, Kegel’s exercise, and pelvic scooting, demonstrated significant improvement in all the outcome measures used.
